# Supplementary material for: Challenges for Precise Subtyping and Sequencing of a H5N1 Clade 2.3.4.4b Highly Pathogenic Avian Influenza Virus Isolated in Japan in the 2022–2023 Season Using Classical Serological and Molecular Methods
Source: Viruses. 2023 Nov 18;15(11):2274. doi: 10.3390/v15112274 (PMC10675786; doi:10.3390/v15112274)

Supplementary Figure S1. PB2 gene (segment 1) phylogenetic tree

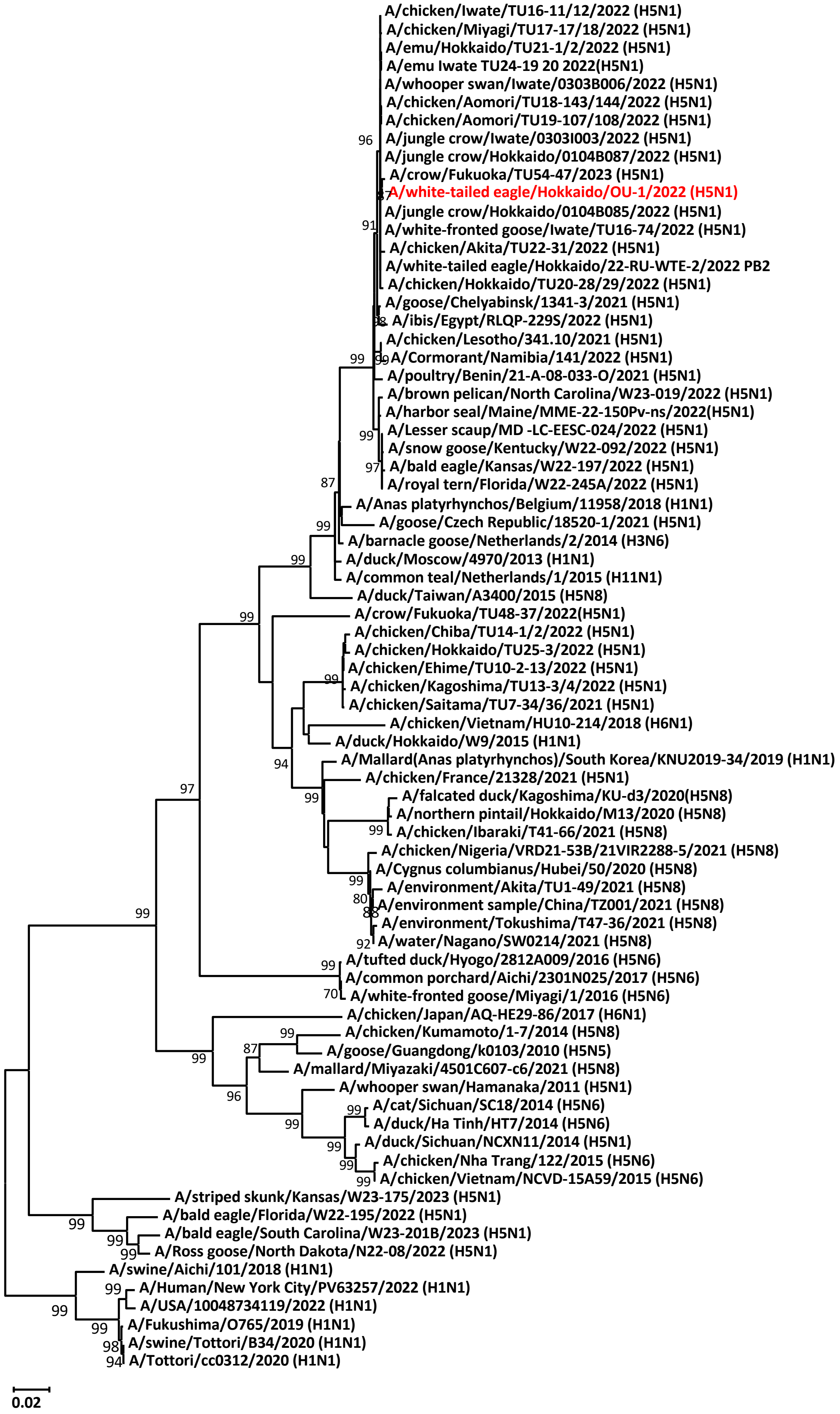

Supplementary Figure S2. PB1 gene (segment 2) phylogenetic tree

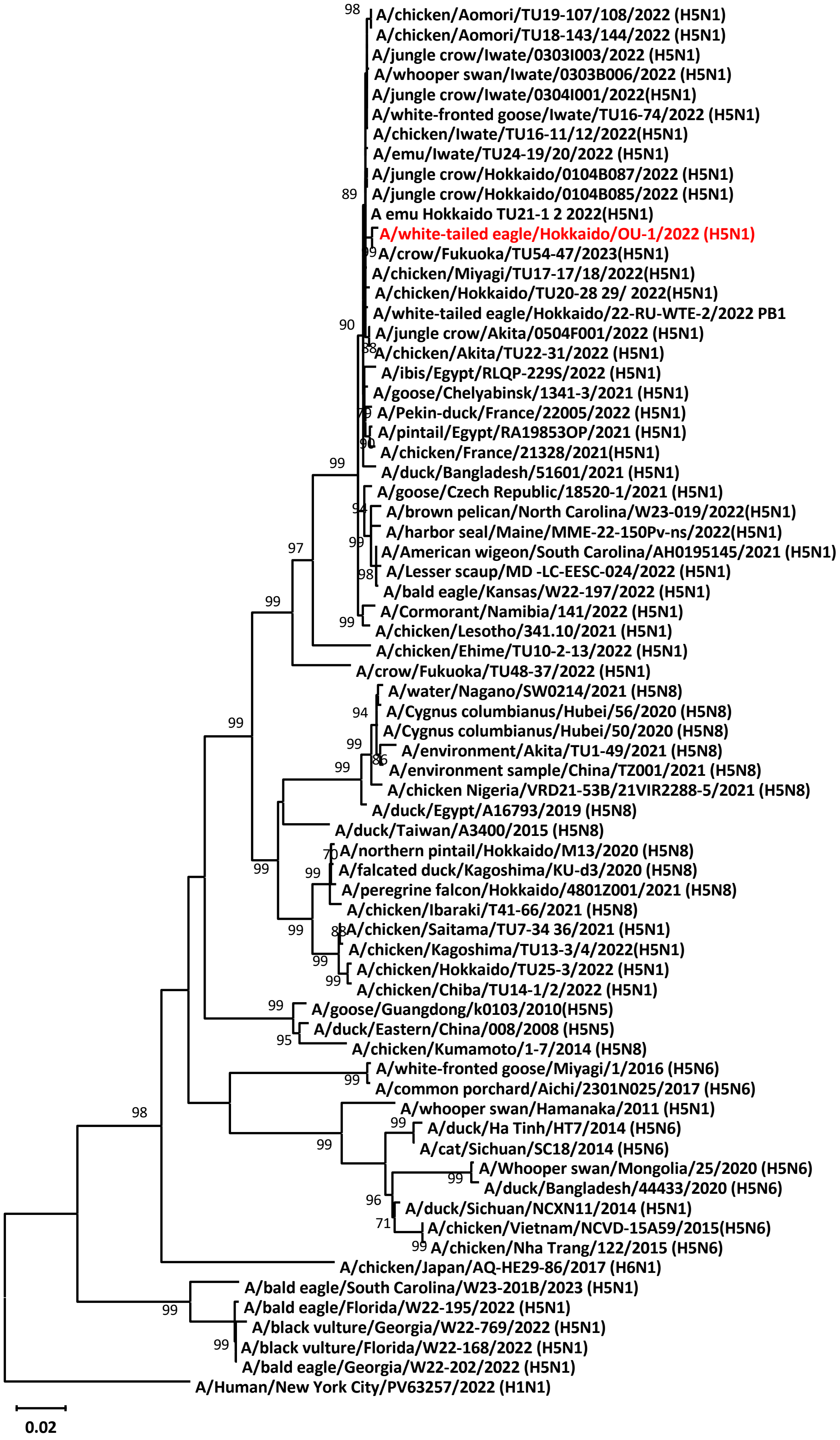

Supplementary Figure S3. PA gene (segment 3) phylogenetic tree

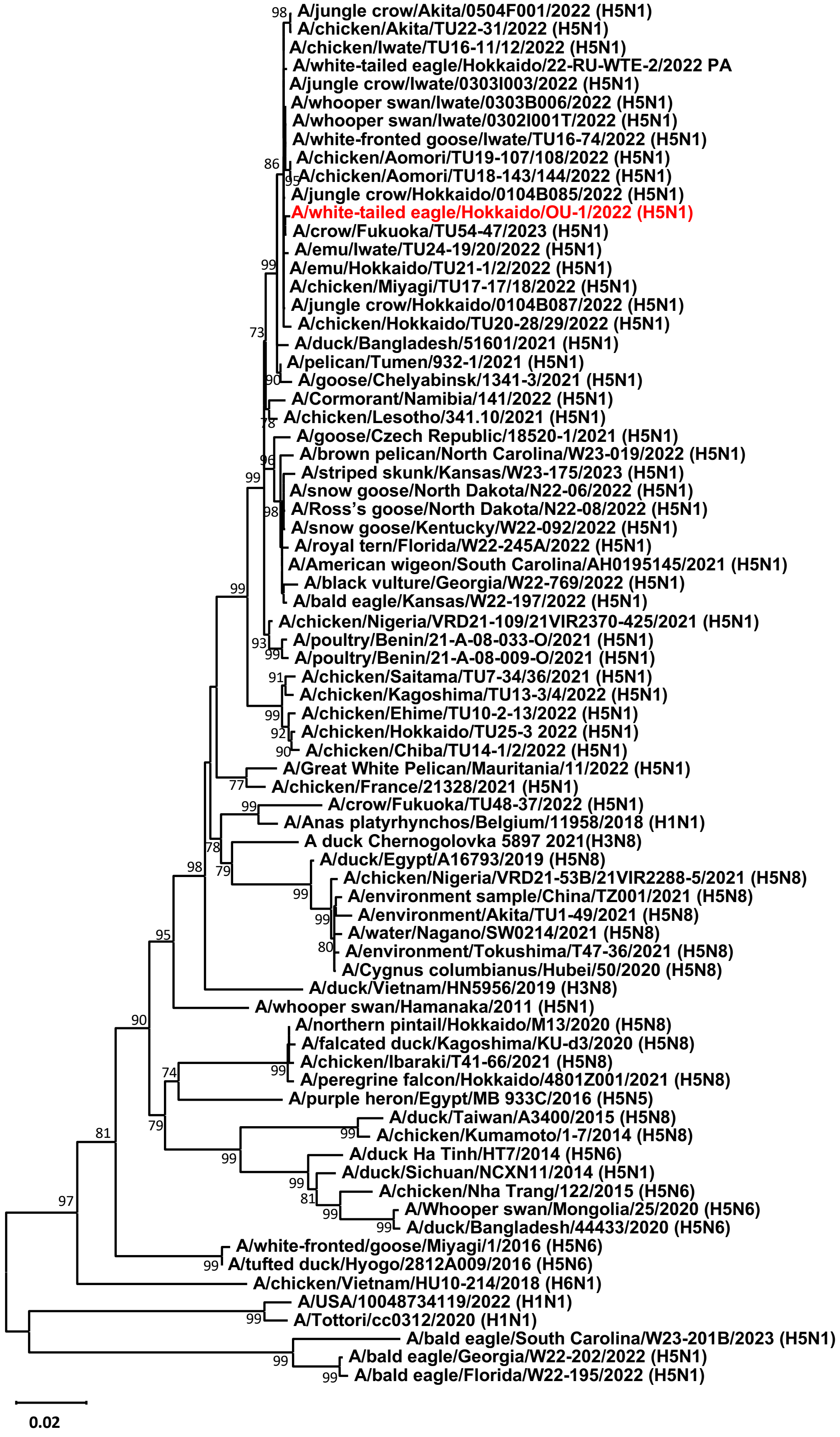

Supplementary Figure S4. NP gene (segment 5) phylogenetic tree

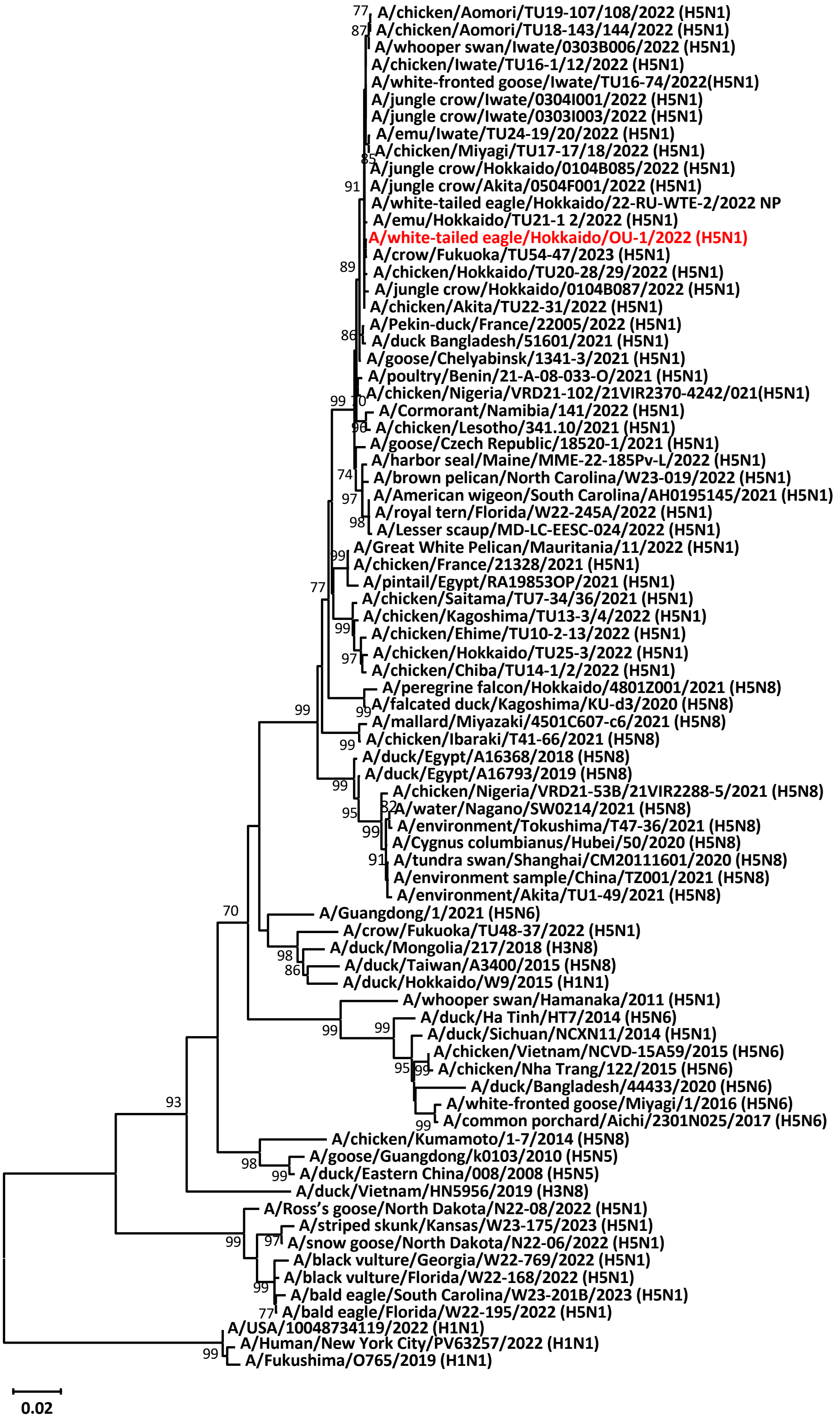

Supplementary Figure S5. NA gene (segment 6) phylogenetic tree

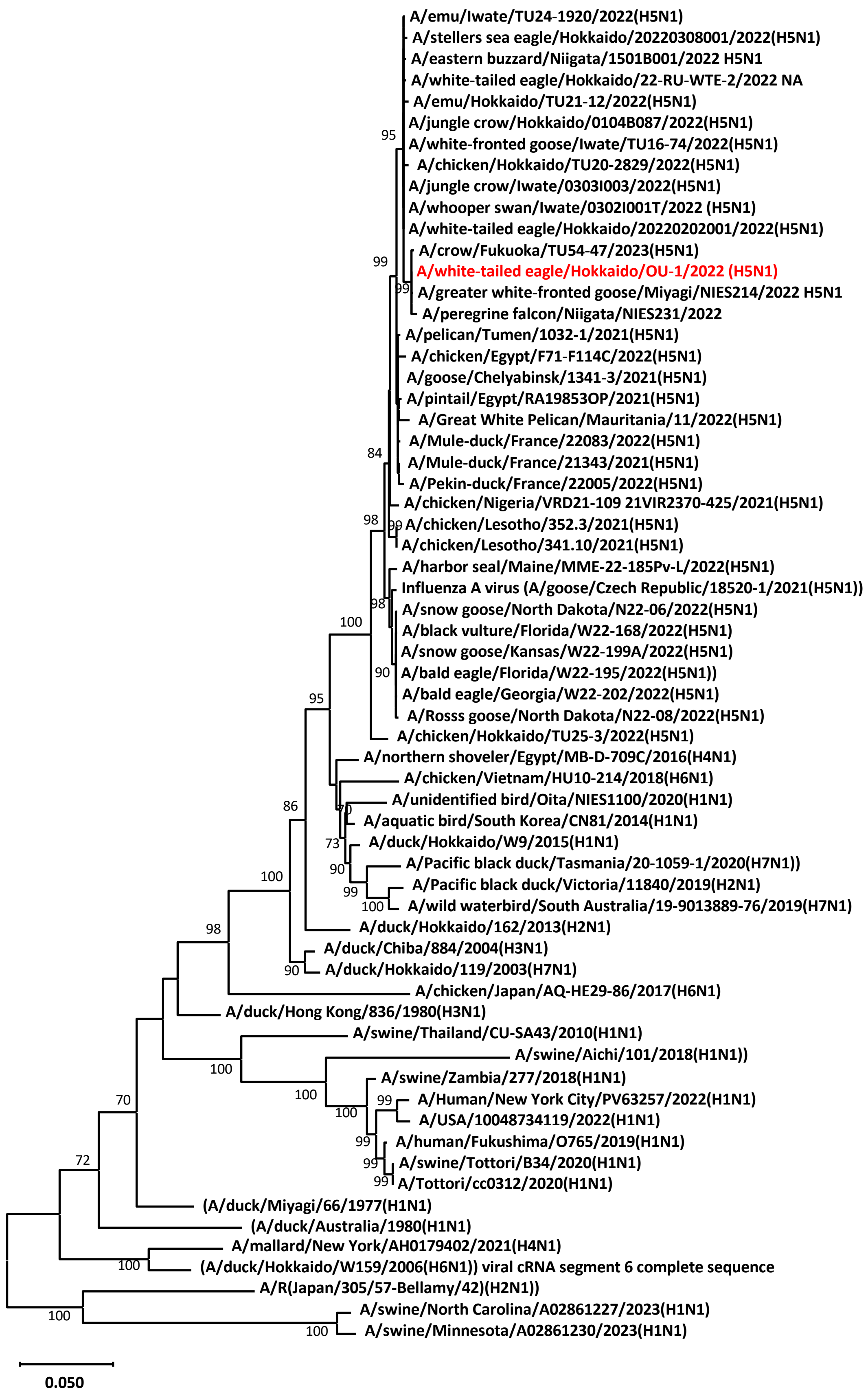

Supplementary Figure S6. M gene (segment 7) phylogenetic tree

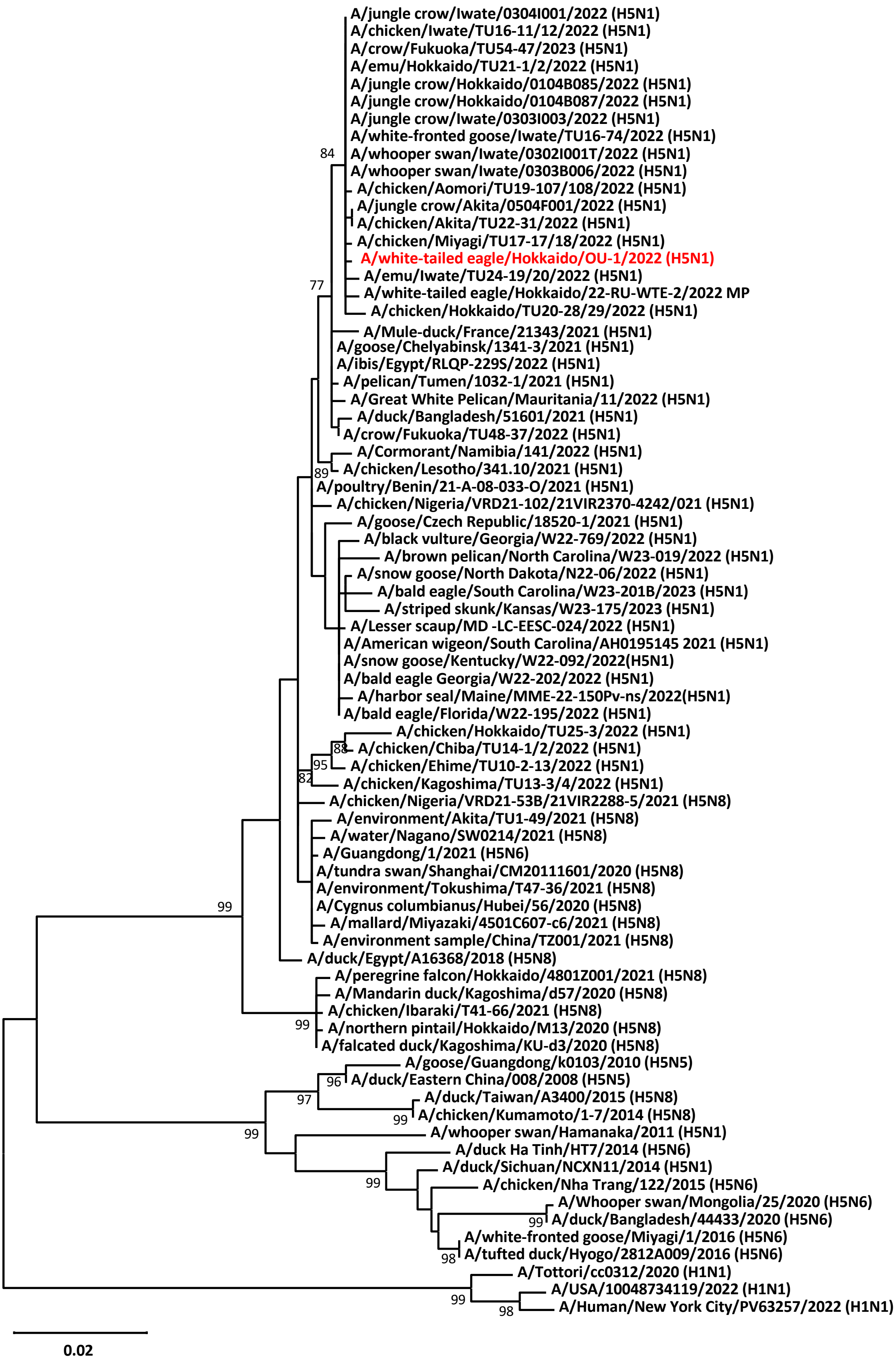

Supplementary Figure S7. NS gene (segment 8) phylogenetic tree

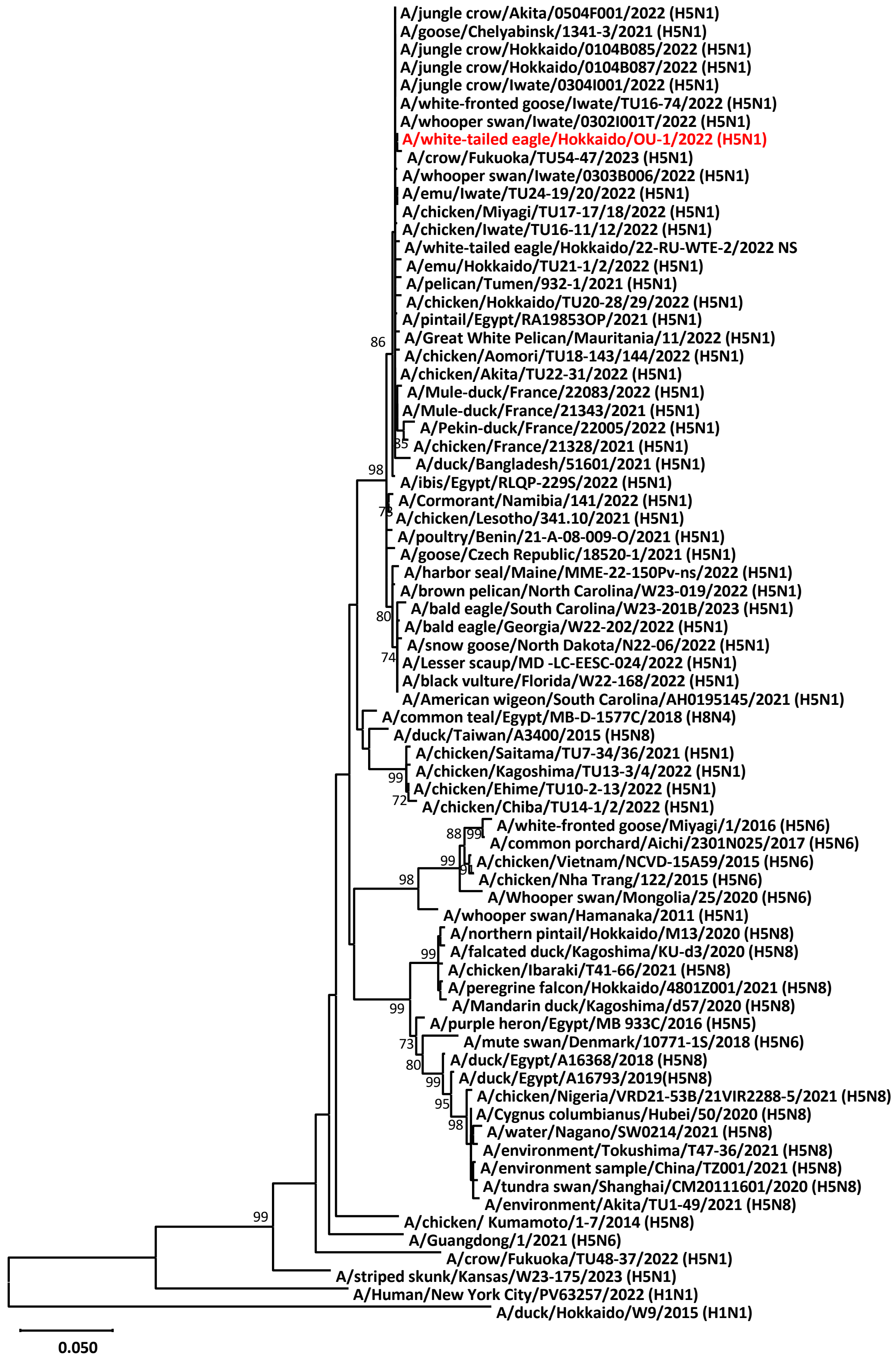

Supplement: Supplementary file 1 [file viruses-15-02274-s001.zip › Supplementary figures.pdf]
