# Supplementary material for: Challenges for Precise Subtyping and Sequencing of a H5N1 Clade 2.3.4.4b Highly Pathogenic Avian Influenza Virus Isolated in Japan in the 2022–2023 Season Using Classical Serological and Molecular Methods
Source: Viruses. 2023 Nov 18;15(11):2274. doi: 10.3390/v15112274 (PMC10675786; doi:10.3390/v15112274)
Supplement: Supplementary file 1 [file viruses-15-02274-s001.zip › Supplementary tables.pdf]

Supplementary Table S1. AIV reference strains used to produce antisera for HA subtyping used in this study (The antisera were kindly provided by OIE Reference Laboratory for AIV, Hokkaido University)

**Strain**	**Subtype**
A/swine/Hokkaido/1/81	H1N1
A/duck/Hong Kong/278/78	H2N9
A/duck/Hokkaido/5/77	H3N2
A/duck/Czechoslovakia/56	H4N6
A/duck/Hong Kong/820/80	H5N3
A/shearwater/South Australia/1/72	H6N5
A/duck/Hong Kong/301/78	H7N2
A/turkey/Ontario/6118/67	H8N4
A/duck/Hong Kong/448/78	H9N2
A/chicken/Germany/N/49	H10N7
A/duck/England/1/56	H11N6
A/duck/Alberta/60/76	H12N5
A/gull/Maryland/704/77	H13N6
A/mallard/Astrakhan/263/82	H14N5
A/duck/Australia/341/83	H15N8

Supplementary Table S2. Percentage nucleotide identify of the genes of A/white-tailed eagle/Japan/OU-1/2022 with its top 4 close relatives derived from BLAST analysis

Gene	Close relatives	Identity (%)
PB2	A/jungle crow/Hokkaido/0104B087/2022 (H5N1)	99.87
	A/jungle crow/Hokkaido/0104B085/2022 (H5N1)	99.87
	A/jungle crow/Iwate/0304I001/2022 (H5N1)	99.87
	A/white-fronted goose/Iwate/TU16-74/2022 (H5N1)	99.87
PB1	A/crow/Fukuoka/TU54-47/2023 (H5N1)	99.67
	A/emu/Hokkaido/TU21-1,2/2022 (H5N1)	99.43
	A/chicken/Miyagi/TU17-17,18/2022 (H5N1)	99.39
	A/jungle crow/Hokkaido/0104B087/2022 (H5N1)	99.39
PA	A/emu/Hokkaido/TU21-1,2/2022 (H5N1)	99.82
	A/chicken/Iwate/TU16-11,12/2022 (H5N1)	99.82
	A/chicken/Miyagi/TU17-17,18/2022 (H5N1)	99.82
	A/crow/Fukuoka/TU54-47/2023 (H5N1)	99.82
HA	A/crow/Fukuoka/TU54-47/2023 (H5N1)	99.94
	A/white-fronted goose/Iwate/TU16-74/2022 (H5N1)	99.77
	A/jungle crow/Iwate/0304I001/2022 (H5N1)	99.77
	A/jungle crow/Hokkaido/0104B085/2022 (H5N1)	99.77
NP	A/crow/Fukuoka/TU54-47/2023 (H5N1)	99.87
	A/jungle crow/Hokkaido/0104B085/2022 (H5N1)	99.81
	A/chicken/Iwate/TU16-11,12/2022 (H5N1)	99.74
	A/chicken/Akita/TU22-31/2022 (H5N1)	99.74
NA	A/crow/Fukuoka/TU54-47/2023(H5N1)	99.86
	Influenza A/peregrine falcon/Niigata/NIES231/2022 (H5N1)	99.86
	A/greater white-fronted goose /Miyagi/NIES214/2022 (H5N1)	99.86
	A/jungle crow/Hokkaido/0104B085/2022 (H5N1)	99.57
M	A/crow/Fukuoka/TU54-47/2023 (H5N1)	99.9
	Influenza A virus (A/chicken/Iwate/TU16-11,12/2022 (H5N1)	99.9
	A/jungle crow/Hokkaido/0104B087/2022 (H5N1)	99.9
	A/whooper swan/Iwate/0303B006/2022 (H5N1)	99.9
NS	Influenza A virus (A/Mule-duck/France/21343/2021 (H5N1)	99.88
	Influenza A virus (A/pelican/Tumen/1032-1/2021 (H5N1)	99.88

	A/goose/Chelyabinsk/1341-3/2021 (H5N1))	99.88
	A/jungle crow/Hokkaido/0104B087/2022 (H5N1)	99.88
